# Engineering a carbohydrate-binding module to increase the expression level of glucoamylase in *Pichia pastoris*

**DOI:** 10.1186/s12934-022-01833-1

**Published:** 2022-05-28

**Authors:** Lige Tong, Huoqing Huang, Jie Zheng, Xiao Wang, Yingguo Bai, Xiaolu Wang, Yuan Wang, Tao Tu, Bin Yao, Xing Qin, Huiying Luo

**Affiliations:** State Key Laboratory of Animal Nutrition, Institute of Animal Sciences, Chinese Academy of Agricultural Sciences, Beijing, 100193 China

**Keywords:** Glucoamylase, Carbohydrate-binding module, Protein expression level, Site-directed mutagenesis, *Pichia pastoris*

## Abstract

**Background:**

Glucoamylase is an important industrial enzyme for the saccharification of starch during sugar production, but the production cost of glucoamylase is a major limiting factor for the growth of the starch-based sugar market. Therefore, seeking strategies for high-level expression of glucoamylase in heterologous hosts are considered as the main way to reduce the enzyme cost.

**Results:**

*Re*Ga15A from *Rasamsonia emersonii* and *Tl*Ga15B-GA2 from *Talaromyces leycettanus* have similar properties. However, the secretion level of *Re*Ga15A was significantly higher than *Tl*Ga15B-GA2 in *Pichia pastoris*. To explore the underlying mechanisms affecting the differential expression levels of glucoamylase in *P. pastoris*, the amino acid sequences and three-dimensional structures of them were compared and analyzed. First, the CBM region was identified by fragment replacement as the key region affecting the expression levels of *Re*Ga15A and *Tl*Ga15B-GA2. Then, through the substitution and site-directed mutation of the motifs in the CBM region, three mutants with significantly increased expression levels were obtained. The eight-point mutant *Tl*GA-M4 (S589D/Q599A/G600Y/V603Q/T607I/V608L/N609D/R613Q), the three-point mutant *Tl*GA-M6 (Q599A/G600Y/V603Q) and the five-point mutant *Tl*GA-M7 (S589D/T607I/V608L/N609D/R613Q) have the same specific activity with the wild-type, and the enzyme activity and secretion level have increased by 4–5 times, respectively. At the same time, the expression levels were 5.8-, 2.0- and 2.4-fold higher than that of wild type, respectively. Meanwhile, the expression of genes related to the unfolded protein responses (UPR) in the endoplasmic reticulum (ER) did not differ significantly between the mutants and wild type. In addition, the most highly expressed mutant, *Tl*GA-M7 exhibits rapidly and effectively hydrolyze raw corn starch.

**Conclusions:**

Our results constitute the first demonstration of improved expression and secretion of a glucoamylase in *P. pastoris* by introducing mutations within the non-catalytic CBM. This provides a novel and effective strategy for improving the expression of recombinant proteins in heterologous host expression systems.

**Supplementary Information:**

The online version contains supplementary material available at 10.1186/s12934-022-01833-1.

## Background

Starch is the most abundant renewable biomolecule in nature except cellulose and hemicellulose [1], and it is widely used in the food, textile, biopharmaceutical and other industries [2]. Enzymes related to amylase-mediated hydrolysis account for 25–30% of the total global enzyme market [3]. High-level expression and secretion of starch-hydrolytic enzymes in heterologous expression systems are particularly important for reducing overall production costs. The *Pichia pastoris* expression system is one of the most widely applied means of producing heterologous proteins [4]. It is cost-effective, produces and secretes heterologous proteins efficiently and with high yield, produces only limited amounts of endogenous proteins, and facilitates easy purification of heterologous proteins [5, 6]. Increasing the expression and secretion of target proteins in *P. pastoris* has always been the top priority for industrial-scale production and application. At present, the research efforts in increasing the yield of recombinant protein mainly focuses on the gene codon optimization, gene copy number increase, promoter selection, molecular chaperone co-expression, protein secretion pathway and methanol metabolism pathway engineering etc. [7]. In addition, the fusion of new modules or the introduction of foreign gene mutations can also improve the expression level of target proteins [8, 9].

The carbohydrate-binding module (CBM) is a non-catalytic auxiliary domain that can promote the binding of a catalytically active domain to carbohydrate [10]. The CBM is usually a β-barrel domain located at the C- or N-terminus of a protein [11]. According to different substrate binding specificity, different CBMs can specifically recognize cellulose, chitin, starch, or glycogen. In the Carbohydrate Active Enzyme (CAZy) database (http://www.cazy.org/), starch binding domain (SBD) is classified as CBMs family, which means CBMs with affinity for starch [12]. The diversity of CBMs is critical promoting the binding of enzymes to specific substrates (especially insoluble substrates), stabilizing enzymes, optimizing both temperature [13–15] and kinetic parameters [16]. By fusing a CBM to the C-terminal region of β-mannanase, Tang et al. reported a significant decrease in *K*_m_ [16]. Although, CBMs have been the subject of much research, most studies have focused on the effects of creating an enzyme-CBM fusion on activity, stability, substrate affinity, and expression level. However, it has not been reported that the expression level affected by the introduction of mutations in the CBMs region.

Glucoamylase (GA) is a starch hydrolase produced mainly by archaea, bacteria and fungi. GAs can hydrolyze glycoside α-1,4 bonds and α-1,6 bonds at the non-reducing ends of starch molecules and were one of the earliest enzyme types applied on a large scale in the food industry [17–20]. Fungal GAs are classified in glycoside hydrolase family 15 [17, 19] and usually contain three different regions, namely a catalytic domain, starch-binding domain, and a glycosylation linker [11, 19]. Depending on the source of GA, the starch binding domain belonging to CBM 20 family was almost exclusively located at the C-terminal of the catalytic domain, while the CBM 21 was linked to the N-terminus of the GA [21, 22]. For example, the CBMs of the CBM 20 family resided at the C terminus of the GA from *Aspergillus niger*, whereas a CBM 21 family member was linked to the N terminus of the GA from *Rhizopus oryzae* [11]. Several expression platforms are currently used for the industrial-scale production of enzymes. Commercially used fungal GAs are usually expressed in *A. niger* [20] or yeast [23]. However, the cost of producing GAs limits their marketability as they are required in starch processing. Therefore, the efficient expression and secretion of GAs in a heterologous host are particularly important aspects of GA production.

Herein, we propose that introducing mutations at the C-terminal region of a CBM can increase the expression and secretion of a recombinant GA in *P. pastoris*. Two GAs with similar properties but distinct secretion levels were heterologously expressed in *P. pastoris*. Segment replacement and site-directed mutagenesis were used to determine the key amino-acid residues that affect secretion. Furthermore, the expression levels of the two GA-CBMs as well as that of genes related to the unfolded protein response (UPR) were measured to explore the underlying mechanism. Finally, the catalytic efficiency of the best mutant was measured using raw corn starch as substrate to assess their potential for industrial applications.

## Results and discussion

### Expression and characterization of *Re*Ga15A in *P. pastoris*

The *Re*Ga15A coding gene from *Rasamsonia emersonii* was cloned and expressed in *P. pastoris* strain GS115. The apparent molecular mass of purified recombinant *R*eGa15A was ~ 75 kDa, which is larger than the theoretical molecular mass of 65.6 kDa. The observed molecular mass difference was likely due to glycosylation that occurred upon expression in *P. pastoris*, which is a common phenomenon for fungal GAs. Upon deglycosylation mediated by an endoglycosidase (Endo H), the recombinant protein had an apparent molecular mass of ~ 70 kDa (Additional file 1: Fig. S1). This difference may be due to the presence of O-glycosylation.

**Fig. 1:**
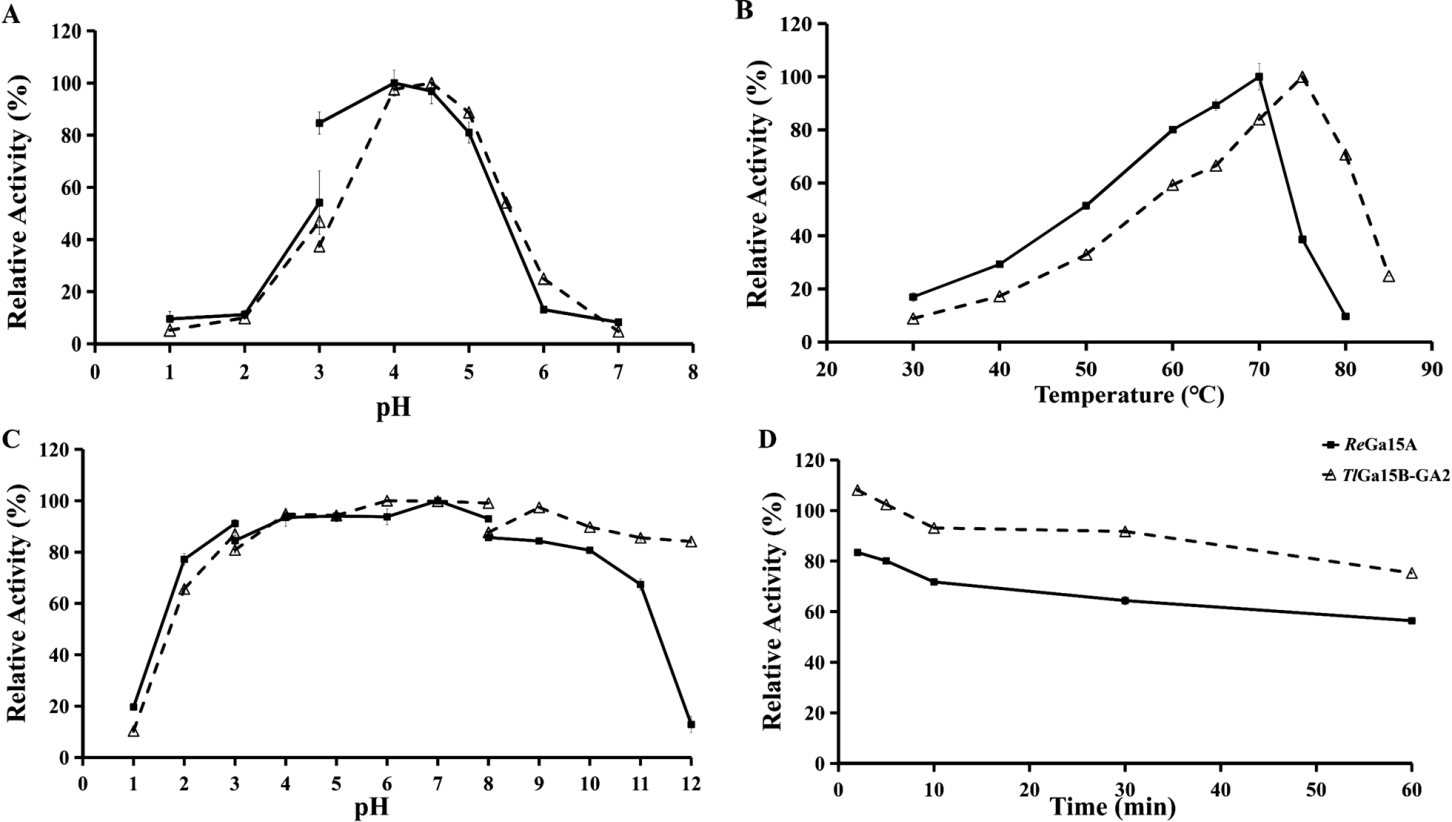
Enzymatic properties of *Re*Ga15A and *Tl*Ga15B-GA2. **a** Effect of pH on enzyme activity. **b** Effect of temperature on enzyme activity. **c** pH stability. **d** Effect of temperature (70℃) on the stability. Each value in the panel represents the means ± SD (n = 3)

Using soluble starch as a substrate, *Re*Ga15A had maximum activity at pH 4.0 and 70 °C (Fig. 1A, B), in agreement with results reported by Bjarne [24]. *Re*Ga15A remained stable over the pH range 2.0–11.0 (Fig. 1C) and at 65 °C (Additional file 1: Fig. S2), and the enzyme retained 64% and 56% of the hydrolytic activity after 30 min and 1 h, respectively, at 70 °C (Fig. 1D). The specific activity of purified recombinant *Re*Ga15A with soluble starch was 1030.2 U/mg and had *K*_m_ and *V*_max_ values of 1.039 mg/mL and 1182 μmoL/min/mg, respectively. Most commercially available GAs are produced mainly from fungal sources, but these commercial GAs have the limitation of relatively low thermostability (55–60 °C) [25]. *Re*Ga15A is the main commercial enzyme at present with high catalytic efficiency and thermostability.

**Fig. 2:**
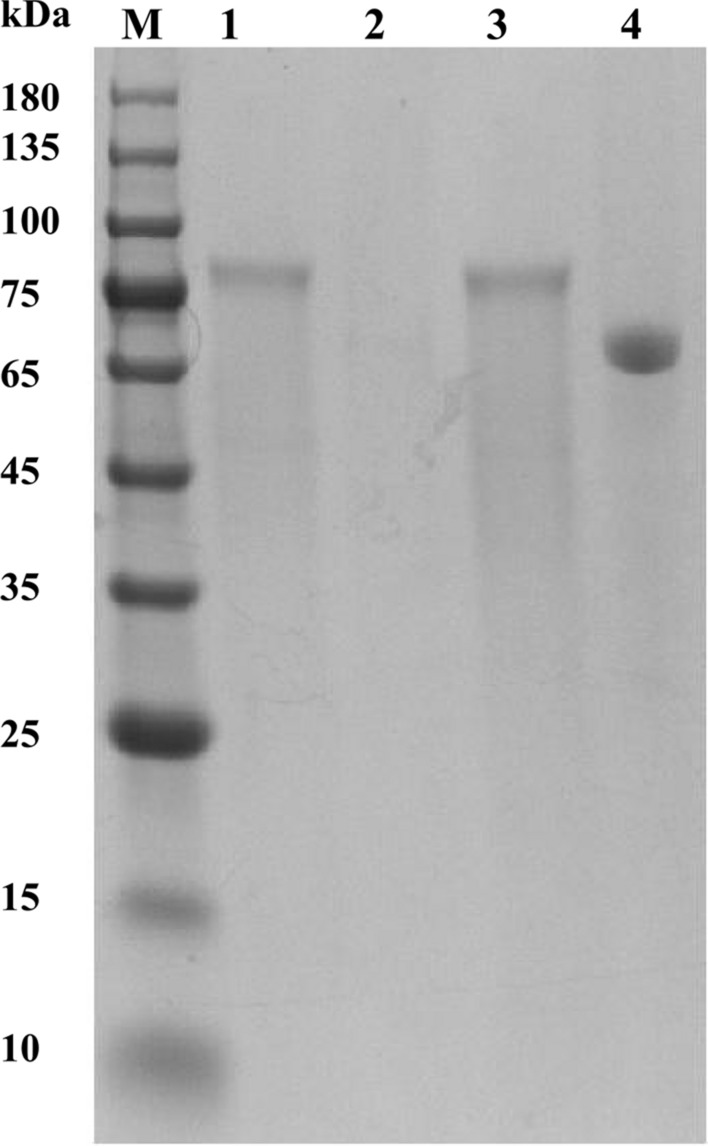
SDS-PAGE analysis of the recombinant *Re*Ga15A and *Tl*Ga15B-GA2. Lane 1, 2, the culture supernatant of transformants *Re*Ga15A and *Tl*Ga15B-GA2; lane 3, 4, the purified *Re*Ga15A and *Tl*Ga15B-GA2

The specific activity of the wild-type (WT) GA from *Talaromyces leycettanus*, namely *Tl*Ga15B-GA2 (hereafter called WT), is 1054.0 U/mg [26], which is similar to that exhibited by *Re*Ga15A; also has similar properties (Additional file 1: Table S3). However, the secretion of these two GAs from *P. pastoris* differs substantially. The activity of *Tl*Ga15B-GA2 and *Re*Ga15A expressed in *P. pastoris* in shake flasks was 9.2 U/mL and 38.6 U/mL, respectively. In addition, based on the results of SDS-PAGE, the quantity of *Re*Ga15A expressed in *P. pastoris* was much greater than that of *Tl*Ga15B-GA2, and indeed no clear bands were observed for *Tl*Ga15B-GA2 (Fig. 2). In addition, the amino-acid sequence identity between *Tl*Ga15B and *Re*Ga15A is 69% (Additional file 1: Fig. S3). Therefore, *Tl*Ga15B-GA2 and *Re*Ga15A could be used to study factors that affect protein secretion from *P. pastoris.*

### Determination of key regions and residues affecting protein secretion

**Fig. 3:**
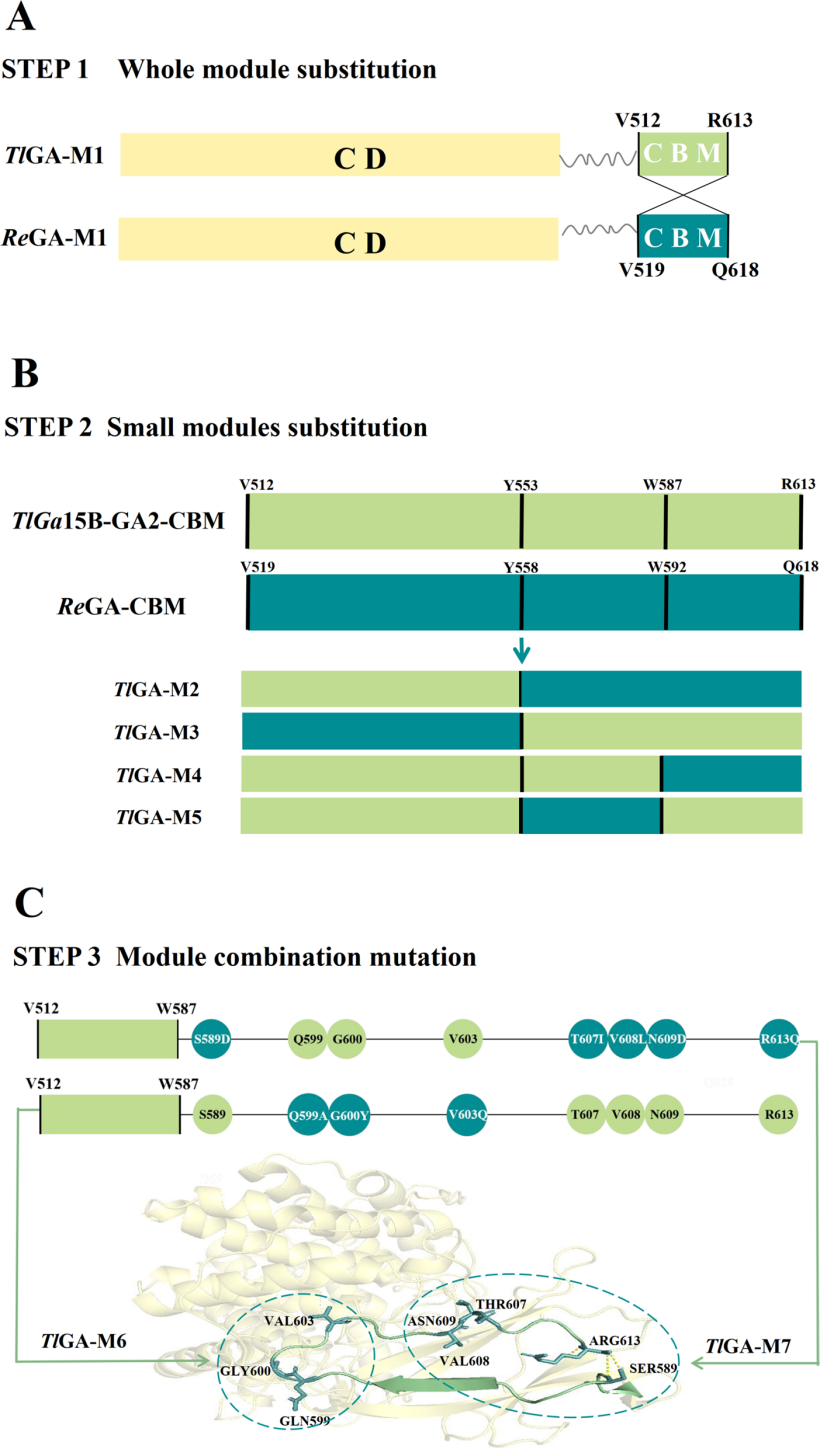
Schematic diagram of the construction of the chimeric mutant. The CD sequences of *Tl*Ga15B-GA2 and *Re*Ga15A are marked in yellow, and the CBM region is marked in green and blue, respectively. **A** CBM substitution. **B** Segment replacement. **C** Construction of combinatorial mutants

**Fig. 4:**
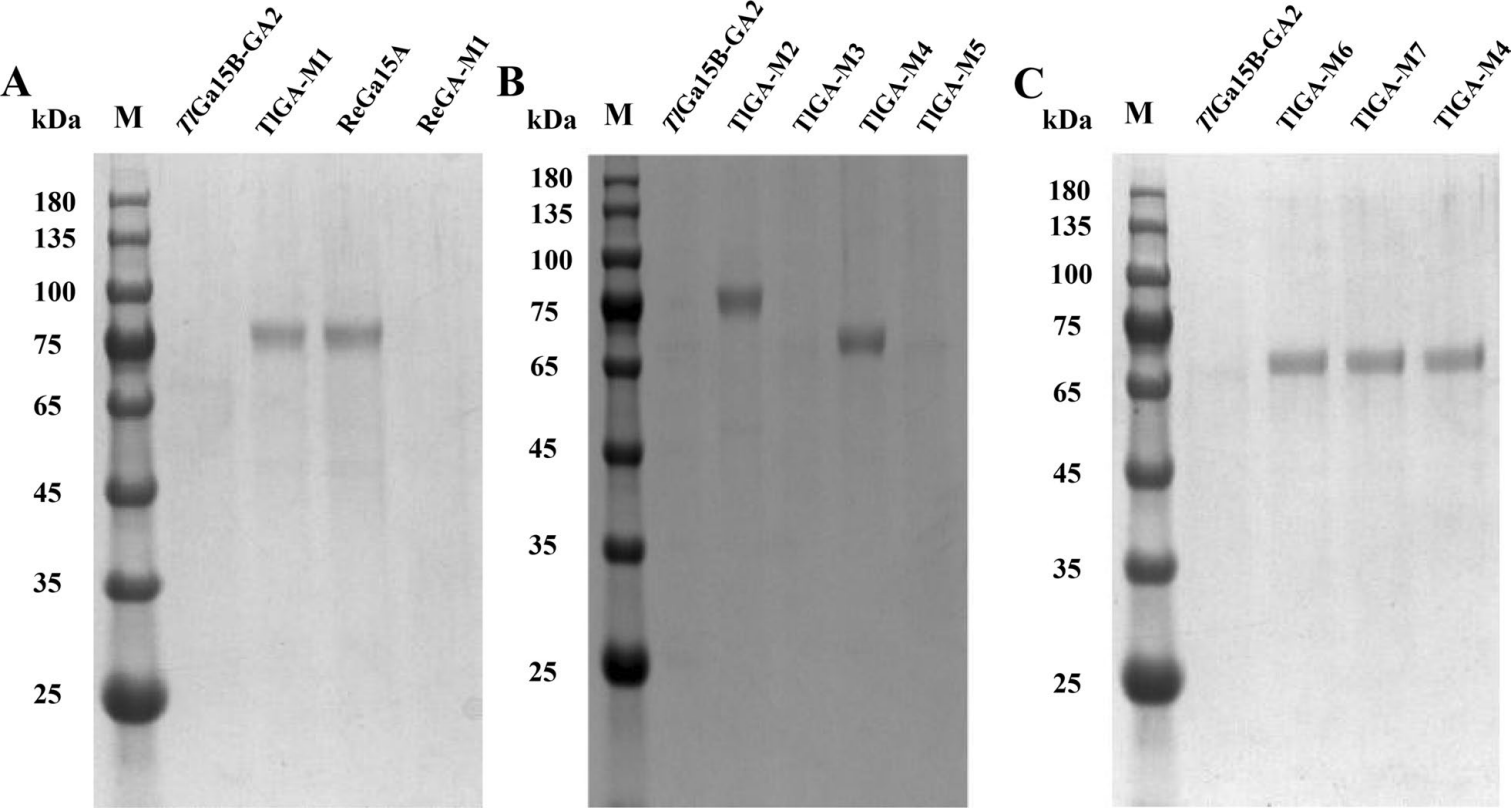
SDS-PAGE analysis of culture supernatants among *Re*Ga15A, *Tl*Ga15B-GA2 and mutants. **A** Secretion analysis of CBM substitution mutants between *Re*Ga15A and *Tl*Ga15B-GA2. **B** Secretion analysis of segment replacement mutants on *Tl*Ga15B-GA2. **C** Secretion analysis of combinatorial mutants on *Tl*Ga15B-GA2

The CBM of *Tl*Ga15B-GA2 (515 V-613R) and *Re*Ga15A (519 V-618Q) were exchanged to explore the key regions affecting expression (Fig. 3A). The two chimeras *Tl*GA-M1 (the CD region of *Tl*Ga15B-GA2 and CBM of *Re*Ga15A) and *Re*GA-M1 (the CD region of *Re*Ga15A and CBM of *Tl*Ga15B-GA2) were obtained. These fusion genes were expressed in *P. pastoris* and secretion level after shake-flask fermentation was compared with that of WT by SDS-PAGE and by measuring enzyme activity. The secretion of *Tl*GA-M1 was significantly greater than that of WT. The activity of the crude enzyme solution of WT increased from 9.2 U/mL to 19.0 U/mL, whereas the secretion level of *Re*GA-M1 was significantly lower (Fig. 4A), with the activity of the crude enzyme solution decreasing from 38.6 U/mL to 12.3 U/mL. Meanwhile, the optimum temperature for the *Tl*GA-M1 mutant decreased from 75 to 65 °C, and both the specific activity and catalytic efficiency were significantly reduced (Table 1).

**Table 1:** The enzyme activity in culture supernatant, specific activity and kinetic parameters of *Tl*Ga15B-GA2 and mutants on soluble starch at the optimum temperature respectively

*Pichia pastoris strain*	Activity (U/mL)	Relative activity (%)	Specific activity (U/mg)	*K*_m_ (mg/mL)	*k*_cat_ (/s)	*k*_cat_/*K*_m_ (mL/s/mg)
*Tl*Ga15B-GA2	9.2±0.1	100	1054.0 ±12.0	0.29 ±0.02	1151.0 ±23.0	3982.6±46.8
*Tl*GA-M1	19.0±0.4	207.3	566.1±6.9	0.87 ±0.08	1260.9 ±35.3	690.2±35.2
*Tl*GA-M2	22.7±0.6	245.6	685.3±5.5	0.38±0.07	741.1±16.2	1955.2±41.9
*Tl*GA-M3	7.2 ±0.3	78.3	807.7 ±6.8	0.41 ±0.11	895.6±13.4	2163.1±33.3
*Tl*GA-M4	36.3 ±0.5	396.4	1004.3±2.0	0.31±0.03	1058.0±17.9	3412.9 ±57.3
*Tl*GA-M5	7.6±0.2	82.6	794.7 ±4.2	0.54±0.17	1047.4±31.7	1939.6 ±42.6
*Tl*GA-M6	37.3±0.6	407.1	1093.8 ±6.1	0.72 ±0.09	1260.9 ±28.1	1759.8 ±29.1
*Tl*GA-M7	38.6 ±0.4	420.6	1112.9 ±8.8	0.38±0.05	1294.3 ±30.4	3378.2 ±31.1

A CBM not only contributes to substrate binding but also stabilizes the molecular structure at high temperatures [27]. A CBM can also affect the stability and activity of the enzyme [15], and Jia et al. were able to obtain a fusion enzyme with greatly increased activity and thermostability by linking a CBM to the C-terminus of cyclodextrin glycosyltransferase [28]. Similarly, the CBM1 module was deleted from a GH5 β-mannanase from *T. leycettanus* JCM12802, resulting in a recombinant enzyme with reduced thermal tolerance at 80 °C [29]. To our knowledge, however, there are few reports on CBM affecting the secretion of the enzyme. Therefore, the result noted above provided a basis for improving the secretion of a GA in a heterologous host.

To explore the key regions or amino-acid residues that could influence enzyme secretion, activity and stability, the CBMs of *Tl*Ga15B-GA2 and *Re*Ga15A were each divided into two domains based on the primary sequences and secondary structures: V512-E552 and N554-Q613 of *Tl*Ga15B-GA2, and V519-A557 and T559-Q618 of *Re*Ga15A. Two chimeric proteins based on *Tl*GA-M1, namely, *Tl*GA-M2 and *Tl*GA-M3, were then obtained by swapping certain sequences. The C-terminal region of *Tl*Ga15B-GA2 (Y553-R613) was then further divided into two subdomains and replaced with the corresponding fragments of *Re*Ga15A, which yielded *Tl*GA-M4 and *Tl*GA-M5 were obtained (Fig. 3B). Each of the four chimeras was expressed individually in *P. pastoris*, and the purified recombinant enzymes were characterized. Among them, the expression of *Tl*GA-M2 and *Tl*GA-M4 was significantly increased compared with WT (Fig. 4B); the activities of the crude enzyme solutions were 28.2 U/mL and 36.3 U/mL respectively, which were 3.1-fold and 4.0-fold higher, respectively, than that of WT *Tl*Ga15B-GA2 (9.2 U/mL). Meanwhile, the C-terminal segment (W587-R613) was identified as the key region affecting secretion and expression of *Tl*Ga15B-GA2.

According to the sequence alignment results and structural analysis of *Tl*Ga15B-GA2 and *Re*Ga15A, the motif (W587-R613) of *Tl*Ga15B-GA2 is oval, in which S589 and R613 are connected by a hydrogen bond, making the region like a "closed" ring. Using *Tl*Ga15B-GA2 as template, eight single point mutations (S589D, Q599A, G600Y, V603Q, T607I, V608L, N609D, R613Q), two-point combination mutations (S589D/R613Q; Q599A/G600Y), and a three-point mutation (TVN607-609ILD) were constructed and expressed. However, compared with the WT, none of the mutants had increased secretion level (Additional file 1: Fig. S4A). The corresponding sites in *Re*Ga15A were also mutated, and the secretion of the mutants did not decrease (Additional file 1: Fig. S4B). Although the secretion of *Tl*Ga15B-GA2 depended on the C-terminal region W587-R613, it was not affected by any single-residue mutation or adjacent binding sites. We speculated that the increase or decrease in secretion was not caused by a change in contribution of any localized amino-acid residue.

According to the structure of *Tl*Ga15B-GA2 motif W587-R613, residues Q599, G600 and V603 and residues S589, T607, V608, N609 and R613 are located at the respective ends of the closed loop (Fig. 3C). The three-point mutant *Tl*GA-M6 (Q599A/G600Y/V603Q) and five-point mutant *Tl*GA-M7 were constructed (S589D/T607I/V608L/N609D/R613Q). Surprisingly, the secretion of mutants *Tl*GA-M6 and *Tl*GA-M7 increased significantly relative to WT (Fig. 4C). The activities of the crude enzyme solutions were 37.3 U/mL and 38.6 U/mL respectively, representing a 4.1-fold and 4.2-fold increase, respectively, compared with WT. These activities were comparable with that of *Tl*GA-M4 (Table 1).

The differential secretion of recombinant proteins between transformants is a common phenomenon in *P. pastoris*, which is called "clonal variability". This change is caused by the insertion of non-specific transgene into genomic DNA, which affects the protein secretion level of *P. pastoris* [30]. In the process of screening positive clones, 48 invertase activities were screened for each mutant to ensure that the secretion of each mutant enzyme was not affected by clonal variability. The results showed that the key region underlying the observed significant difference in secretion between *Tl*Ga15B-GA2 and *Re*Ga15A was the motif at the C-terminus of the CBM, and the difference was not due to individual residues but rather through a small-scale combination of residues in different regions.

### Characterization of *Tl*GA-M4, *Tl*GA-M6 and *Tl*GA-M7 expressed in *P. pastoris*

To better understand the effects of different motifs within the *Re*Ga15A CBM sequence on *Tl*Ga15B-GA2, enzymatic properties were determined for mutants *Tl*GA-M4, *Tl*GA-M6 and *Tl*GA-M7 expressed in *P. pastoris*. Under optimum reaction conditions for each enzyme (70 or 75 °C, pH 4.5), the kinetics were studied with soluble starch as substrate. The specific activities of *Tl*GA-M4, M6, M7 were 1004.3 U/mg, 1093.8 U/mg and 1112.9 U/mg, respectively, and these values were consistent with those for *Tl*Ga15B-GA2 (1054.0 U/mg). Surprisingly, compared with *Tl*Ga15B-GA2, *Tl*GA-M6 had decreased substrate affinity (a higher *K*_m_ value) and catalytic efficiency (*k*_cat_/*K*_m_) (Table 1). Previous researchers also analyzed the structure of the saccharifying enzyme of *A. niger* [31], revealing two substrate-binding sites comprised of two residues each. The results of our molecular dynamics simulations revealed that the root mean square fluctuation (RMSF) values for two of the corresponding residues (W540 and Y553) of mutant *Tl*GA-M6 were significantly lower than those of WT and other mutants (Additional file 1: Fig. S5B). In general, RMSF measures the amplitude of atomic motion during simulation. A lower RMSF value means less flexibility [32]. We speculate that the low flexibility of these two substrate-binding residues within the polypeptide chain of the starch-binding domain led to the observed low binding affinity for starch. This caused an increase in the *K*_m_ of value for mutant *Tl*GA-M6.

**Fig. 5:**
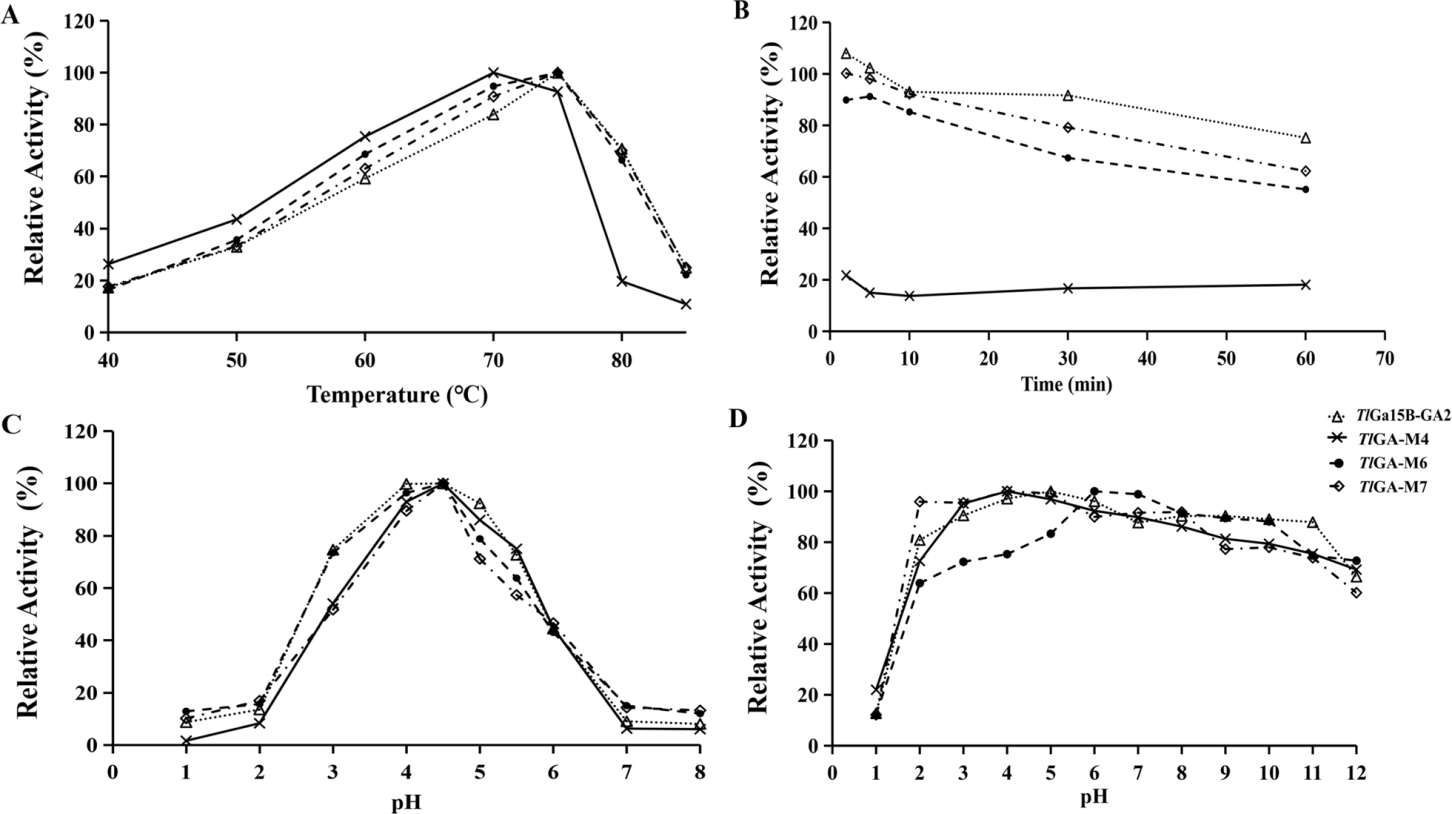
Effect of pH and temperature on the activity and stability of purified recombinant *Tl*Ga15B-GA2 and mutants.** a**: Effect of temperature on the activity.** b**: Effect of temperature (70℃) on the stability.** c**: Effect of pH on the activity.** d**: Effect of pH on the stability

Other enzymatic characteristics were also analyzed. The optimal temperature for mutant *Tl*GA-M4 activity was 70 °C, which was 5 °C lower than that of WT, *Tl*GA-M6, and *Tl*GA-M7 (75 °C) (Fig. 5A). The thermostability of *Tl*GA-M4 also decreased significantly compared with WT, as the remaining enzyme activity after 30 min of incubation at 70 °C was only 16.7%, whereas that measured for *Tl*Ga15B-GA2 was 96.7%. However, the thermostabilities of *Tl*GA-M6 and *Tl*GA-M7 were almost the same as that of the *Tl*Ga15B-GA2 (Fig. 5B). This result indicated that the eight-point combination of mutations in mutant *Tl*GA-M4 had a negative effect on the stability of *Tl*Ga15B-GA2, but neither the three-point nor five-point mutations had an effect. Increasing the global root mean square deviation (RMSD) of a protein has the effect of increasing the flexibility of the enzyme structure and thus reducing its thermostability [33]. Compared with WT *Tl*Ga15B-GA2 and mutants *Tl*GA-M6 and *Tl*GA-M7, the RMSD value for mutant *Tl*GA-M4 after 15 ns equilibrium was higher, indicating that the overall structural stability of the protein was reduced by the eight-point mutation (Additional file 1: Fig. S5A). The average structure extracted in the last 5 ns of the equilibrium state simulated by molecular dynamics was used as a reference. The WT enzyme contains a disulfide bond (C132/C492), but this bond does not exist in *Tl*GA-M4 (Additional file 1: Fig. S5C, D). Previous experiments have shown that the introduction of a disulfide bond at this position has a positive effect on the overall thermostability of the enzyme [34]. For the three mutants *Tl*GA-M4, M6, and M7, the highest activity was attained at pH 4.5 (Fig. 5C), and each retained > 60% of initial activity after 60 min of incubation at 37 °C over a pH range of 2.0–12.0; these results were similar to those of *Tl*Ga15B-GA2 (Fig. 5D).

### Expression analysis of the GA gene and UPR-related proteins

**Fig. 6:**
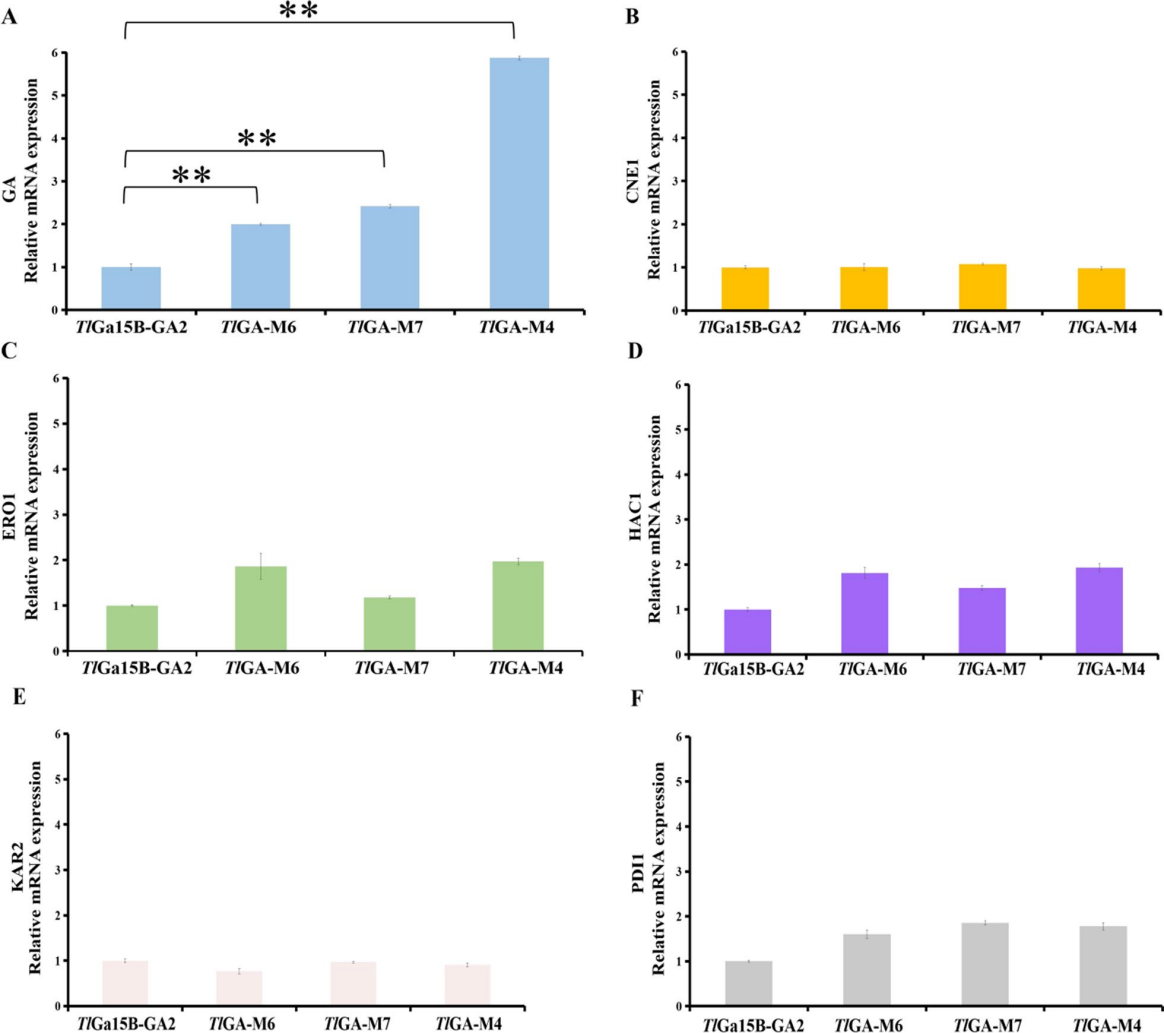
Use qRT-PCR to determine the relative expression levels of WT and mutants and the relative expression of genes related to quality control of UPR and ER. *CNE1*: calnexin (ER chaperone); *ERO1*: Pdi oxidase; *HAC1*: UPR activated transcription factor; *KAR2*: ER chaperone; *PDI1*: protein disulfide isomerase. The experiment is performed in at least triplicate, and the error bars represent the standard deviation. The 2.^−ΔΔCT^ method was used to determine the relative expression, and the expression level of *ARG4* in the strain was used as a reference. **: P value < 0.01 (t test)

In eukaryotic cells, high expression of exogenous or endogenous proteins will lead to the accumulation of unfolded new peptides in the endoplasmic reticulum (ER), which initiates a stress response of the ER, i.e., the UPR, owing to a disorder of folding or secretion. Therefore, to maintain ER homeostasis, UPR is activated [35]. To further understand the expression of GA genes in *P. pastoris*, we performed quantitative reverse transcription-PCR (qRT-PCR) analysis to analyze the levels of mRNAs encoded by genes involved in UPR. Compared with *Tl*Ga15B-GA2, the relative expression levels of GA genes of *Tl*GA-M6, *Tl*GA-M7 and *Tl*GA-M4 was increased by 2.0-fold, 2.4-fold, and 5.8-fold, respectively (Fig. 6A). Thus, the relative expression of the mutant GAs was substantially higher than that of WT which means that the mutation has an impact on the expression of GA.

Considering that the expression of UPR-related genes containing calnexin (*CNE1*), Pdi oxidase (*ERO1*), UPR activated transcription factor (*HAC1*), ER chaperone (*KAR2*) and protein disulfide isomerase (*PDI1*) could reflect the ER stress [8, 35–38]. qRT-PCR analysis was used to determine the relative expression levels of these genes between WT and mutants (Fig. 6B–F). The heterologous expression of WT or any mutants in *P. pastoris* did not cause an increase in the expression of UPR-related genes. The expression levels of *KAR2* and *CNE1* did not change, whereas expression of *ERO1*, *HAC1* or *PDI1* in the mutants was slightly upregulated but not significantly.

Based on these results, we speculate that mutations in the C-terminus of the starch-binding domain can promote the sorting and folding of functionally enhanced enzyme polypeptides in the ER. Notably, even if the expression of any particular mutant GA gene was significantly upregulated compared with WT, this did not substantively activate the high level of UPR. A reduced UPR may also lead to weaker protein degradation in the ER. Here, the secretion of the recombinant mutants *Tl*GA-M4, *Tl*GA-M6, and *Tl*GA-M7 was ~ fourfold that of *Tl*Ga15B-GA2, and the folding and sorting efficiency in the ER was higher. This study provides an effective method to improve the stability and folding of heterogenous proteins in the ER, thereby increasing the production of exogenous GAs in *P. pastoris.*

### Scanning electron microscopy (SEM) analysis

**Fig. 7:**
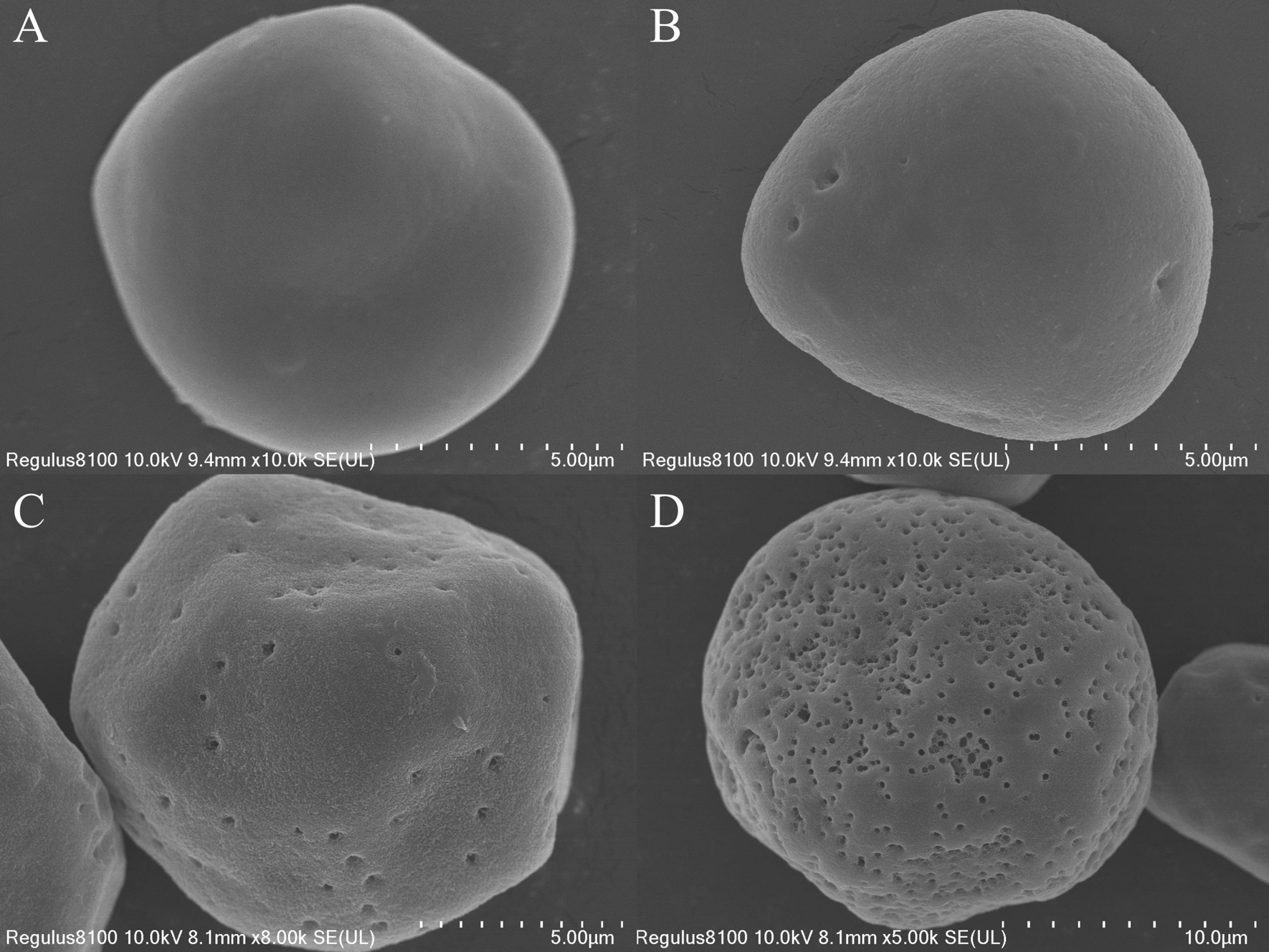
Scanning electron micrograph images of raw corn starch after incubation with *Tl*GA-M7 at 50 °C, for **A** 0, **B** 6, **C** 12, and **D** 24 h

Porous starch has received much attention due to its abundant micropores and excellent adsorption performance [39]. In the fields of food, medicine, chemicals, cosmetics, and agriculture, among others, porous starch can be used as an adsorbent [40, 41] Especially in the food industry, porous starch is an ideal slow-release material for adsorbing spices, sweeteners, acid seasonings, enzymes, and seasonings [42]. Raw corn starch hydrolyzed by a saccharifying enzyme can be used to prepare porous starch. Therefore, we examined whether our best mutant GA, namely *Tl*GA-M7, could hydrolyze corn starch effectively. Scanning electron microscopy clearly revealed that *Tl*GA-M7 could effectively hydrolyze corn starch particles (Fig. 7). The untreated (control) corn starch particles were visualized as irregular polygons with obvious edges and remained intact and smooth (Fig. 7A). With extended hydrolysis time (6–24 h) with *Tl*GA-M7, however, the pores on the starch surface gradually increased in both number and size (Fig. 7B–D). Notably, the starch granules remained intact after 24 h of hydrolysis with *Tl*GA-M7, and the temperature used in the reaction (50 °C) was lower than the gelatinization temperature of corn starch (65–72 °C) [43, 44]. The ability of *Tl*GA-M7 to efficiently hydrolyze corn starch demonstrates that this mutant GA has great potential for applications in the food and fermentation industries.

## Conclusions

In summary, the CBM region of glucoamylase not only affects the key enzymatic characteristics such as protein activity, stability and substrate affinity, but also plays an important role in the expression and secretion level. Moreover, the key amino-acid residues affecting the expression and secretion were identified by segment replacement and site-directed mutagenesis. The enzymatic properties of the mutant *Tl*GA-M7 make it a promising candidate for application in industrial food saccharification. Our work provides a novel and effective strategy for improving the expression of recombinant proteins in heterologous host expression systems by engineering a CBM.

## Materials and methods

### Strains, vectors, chemicals and substrates

*Escherichia coli* TransI-T1 was obtained from TransGen (Beijing, China) and used as the host for plasmid construction. *Pichia pastoris* GS115 and plasmid pPIC9 from Invitrogen (Carlsbad, CA, USA) were used for gene expression. Plasmid extraction kits were purchased from TIANGEN (Beijing, China), and restriction endonucleases were purchased from New England Biolabs (Ipswich, MA, USA). The DNA isolation kit and DNA polymerase pfu were purchased from TIANGEN. The DNA purification kit and LA Taq DNA polymerase were purchased from TaKaRa (Tsu, Japan). FastPfu Fly DNA polymerase, assembly kit, and fast mutagenesis system were purchased from TransGen. All other chemicals were of analytical grade and commercially available. Substrates such as soluble starch were purchased from Sigma-Aldrich (St. Louis, MO, USA).

### Gene cloning and construction of recombinant plasmids

The GA gene *Re*Ga15A from *R. emersonii* (GenBank: CAC28076.1) without the signal-peptide coding sequence was chemically synthesized by GENEWIZ (Jiangsu, China), and the codons were optimized based on the codon preference of *P. pastoris*. The gene fragment was amplified using specific primers ReGA-F (AAGCTTACGTAGAATTCCGAGCGCCCGTTGCAGCG) and ReGA-R (GAATTAATTCGCGGCCGCTTATCTCACTGCC). The PCR product was digested with *Eco*R I and *Not* I and cloned into pPIC9 that had been digested with the same restriction endonucleases for subsequent expression. The recombinant plasmid pPIC9-*Tl*Ga15B-GA2 of the GA mutant was constructed during a previous study[26]. The gene fragments encoding the chimeric proteins were then obtained by overlap extension PCR using plasmids pPIC9-*Re*Ga15A and pPIC9-*Tl*Ga15B-GA2 as templates and specific primers (Additional file 1: Table S1). Site-directed mutagenesis using pPIC9-*Tl*Ga15B-GA2 as the template was performed by PCR according to the manufacturer's instructions (TransGen) (Additional file 1: Table S1). The PCR products were digested with DMT Enzyme from TransGen (Beijing, China) to remove the methylated plasmid template. The *E. coli* TransI-T1 competent cells were transformed with individual constructs by the heat-shock method, and positive clones were verified by DNA sequencing.

### Design, sequencing, and structure analysis of mutants

Amino-acid sequences related to *Tl*Ga15B-GA2 and *Re*Ga15A were collected by BLAST (http://www.ncbi.nlm.nih.gov/BLAST/), and selected sequences were aligned using ClustalW. Homology modeling of *Tl*Ga15B-GA2 and *Re*Ga15A was conducted with SWISS-MODEL, and the structure 6FHV of *Penicillium oxalicum* GA [45] was used as a template. The amino-acid sequence of 6FHV is 57.79% and 53.29% identical to that of *Tl*Ga15B-GA2 and *Re*Ga15A, respectively. Each modeled structure was optimized to minimize energy and eliminate spatial conflicts by Discovery Studio (DS). Based on the sequence and structure analysis, the CBM substitutions between *Tl*Ga15B-GA2 and *Re*Ga15A were conducted. At the C-terminus (W587-R613) of *Tl*Ga15B-GA2, eight residues differ from *Re*Ga15A, and the single-point mutants, two-point combinatorial mutant, three-point mutant, and five-point mutant were constructed and expressed in *P. pastoris*.

### Expression and purification of recombinant GAs and mutants

Recombinant proteins were expressed using the protocol of Hua et al. [46]. The corresponding positive transformants were screened according to the GA activities described below. Transformants with the highest GA activity were selected for growth in 300 mL buffered glycerol complex medium (BMGY). After 48 h incubation at 30 °C and 220 rpm, the cells were harvested by centrifugation for 10 min at 12,000 × *g* and resuspended in 200 mL buffered methanol complex medium (BMMY) containing 0.5% (v/v) methanol and for subsequent fermentation. After 48 h of cultivation at 30 °C and 220 rpm, the culture supernatants were harvested at 12,000 × *g* for 10 min. Cell-free supernatants were concentrated using an ultrafiltration membrane with a molecular cut-off of 10 kDa (Vivascience, Hannover, Germany). Buffer was replaced with 20 mM McIlvaine buffer (pH 6.3) via dialysis overnight. For protein purification, the crude enzymes were loaded separately onto a HiTrapTM Q Sepharose XL 5-mL FPLC column (GE Healthcare, Uppsala, Sweden), which was equilibrated with 20 mM McIlvaine buffer (pH 6.3). Proteins were eluted with a linear NaCl gradient of 0 to 1.0 M. The purity and apparent molecular mass of purified GAs were estimated by SDS-PAGE [47]. Endo-β-N-acetylglucosaminidase H (Endo H; New England Biolabs) was applied to remove N-glycans. Protein concentrations were determined by the Bradford method using bovine serum albumin as the standard [26].

### GA activity assay and biochemical characterization of purified *Tl*Ga15B-GA2, *Re*Ga15A, and mutants

The GA activity assay for *Tl*Ga15B-GA2, *Re*Ga15A, and mutants followed the protocol of Tong et al. [26]. One unit (U) of enzyme activity was defined as the amount of enzyme that released 1 μmol glucose per minute. The optimum temperature for each of the recombinant enzymes *Tl*Ga15B-GA2 and *Re*Ga15A and their respective mutants was determined in 100 mM McIlvaine buffer at pH 4.5 or 4.0 over the temperature range 30–80 °C. To assess the thermostability of GAs, samples were incubated at different temperatures (50–75 °C) for different periods (0–60 min), and residual activity was measured as described above. At the optimum temperature, the enzyme solution was incubated in 100 mM glycine-hydrochloric acid (pH 1.0–3.0), citrate-Na_2_HPO_4_ (pH 3.0–8.0), and glycine–NaOH (pH 8.0–12.0) buffer, and the optimum pH was determined. In the absence of substrate, each GA was incubated for 1 h at 37 °C in McIlvaine buffer at pH 4.5 or 4.0, and then residual activity was determined as noted above at different pH values (1.0–12.0). Kinetic parameters were estimated for 15 min at pH 4.5 or 4.0 and at the optimal temperature using 1.0–10.0 mg/mL soluble starch as substrate. Lineweaver–Burk plots were used to calculate *K*_m_ and *V*_max_ values.

### Molecular dynamics simulation

To predict the mechanism of the altered kinetics and thermostability of mutants, molecular dynamics simulations of the model structures of *Tl*Ga15B-GA2, *Tl*GA-M4, *Tl*GA-M6 and *Tl*GA-M7 were performed using the AMBER14 package and the ff99SB force field as described by Kirschner et al. [48]. The closest distance between the periodic box and atom was set as 12 Å, and the time step was set to 2 fs. Before simulation, hydrogen atoms were added, and any water molecules that did not interact with the protein were removed, and 20 mM sodium chloride was added to neutralize the charge in the system. The conjugate gradient method was used for energy minimization with α-carbon atom restriction. The energy was minimized again without restricting the atoms, and the temperature was raised from 0 to 300 K. Simulation was carried out at 300 K for 20 ns. The WT and mutants were subjected to simulation three times. The molecular dynamic trajectories were collected every 2 ps for further analysis. The interactions between residues were analyzed using Discovery studio (Accelrys, San Diego, USA), and RMSF and RMSD values were shown after simulation.

### qRT-PCR

RNA was isolated from recombinant *P. pastoris* cells after 48 h of methanol induction at 30 °C using TRIzol Reagent (Invitrogen). The reverse transcription assay was performed with the First Strand cDNA Maxima Synthesis kit (TOYOBO, Shanghai, China). The relative expression levels of genes involved in the UPR were measured using qRT-PCR with a 25-μL reaction system, including 12.5 μL TransScript Green One-Step qRT-PCR SuperMix (TransGen), PCR Forward Primer (0.25 μL, 10 µM), PCR Reverse Primer (0.25 μL, 10 µM), 1 μL cDNA, and 11 μL double-distilled water. The QuantStudio 6 Flex Real-Time PCR System (Applied Biosystems, San Diego, CA, USA) was used to perform the PCR using the following 45-cycle thermal program: 95 °C for 2 min, 95 °C for 10 s, and 60 °C for 1 min. The *P. pastoris* gene *ARG4* (XP_002490047.1) was used as an internal reference gene, and all primers used for qRT-PCR are listed in Additional file 1: Table S2.

### Raw corn starch degradation assay and scanning electron microscopy

To measure the ability of *Tl*GA15B-M7 to hydrolyze raw corn starch, 0.1 mL crude enzyme solution and 0.9 mL of 1% raw corn starch were mixed in phosphate-buffered saline. The reaction was carried out at 50 °C and pH 4.5 for 0, 6, 12, or 24 h. After centrifugation at 8000 × *g* for 10 min, the digested starch granules were washed three times with 95% ethanol. After drying at 37 °C, each sample was analyzed with a Regulus 8100 scanning electron microscope [49].

## Supplementary Information

**Additional file 1: Table S1.** Primers used in this study. **Table S2.** qRT-PCR primers used in this study. **Table S3.** Comparison of enzymatic properties of TlGa15B-GA2 and ReGa15A. **Fig. S1.** SDS-PAGE analysis of the recombinant ReGa15A. **Fig. S2.** The thermostability of the purified recombinant ReGa15A. **Fig. S3.** Multiple sequence alignment of ReGa15A (GenBank: CAC28076.1) and TlGa15B. **Fig. S4.** SDS-PAGE analysis of the recombinant ReGa15A, TlGa15B-GA2 and mutants. **Fig. S5.** MD simulation analysis of TlGa15B-GA2 and mutants.

## Data Availability

All data generated or analyzed during this study are included in this published article and its additional files.

## Abbreviations

CBM: Carbohydrate-binding module
SBD: Starch binding domain
SDS-PAGE: Sodium dodecyl sulfate–polyacrylamide gel electrophoresis
*K*_m_: Michaelis constant.
*V*_max_: Maximum reaction rate.
*k*_cat_: Turnover number
GH: Glycoside hydrolase
WT: Wild type
PDB: Protein Data Bank
DNS: 3, 5-Dinitrosalicylic acid
MD: Molecular dynamics
SEM: Scanning electron microscopy
GA: Glucoamylase
UPR: Unfolded protein response
