# Supplementary material for: Engineering a carbohydrate-binding module to increase the expression level of glucoamylase in Pichia pastoris
Source: Microb Cell Fact. 2022 May 28;21:95. doi: 10.1186/s12934-022-01833-1 (PMC9148494; doi:10.1186/s12934-022-01833-1)

**Table S1** Primers used in this study.

| **Primers** | **Sequences (5'→3')*** |
| --- | --- |
| *Re*GA-F | AAGCTTACGTAGAATTCCGAGCGCCCGTTGCAGCG |
| *Re*GA-R | GAATTAATTCGCGGCCGCTTATCTCACTGCC |
| *Tl*GAM1-F1 | TCGCCACCGGCAAACGTGGCTGTGACCTTC |
| *Tl*GAM1-R1 | TTAATTCGCGGCCGCTTATCTCACTGCCAA |
| *Tl*GAM1-F2 | GCTTACGTAGAATTCGCACCACATCCCACGGAA |
| *Tl*GAM1-R2 | GAAGGTCACAGCCACGTTTGCCGGTGGCGA |
| *Tl*GAM2-F1 | GCTTACGTAGAATTCGCACCACATCCCACGGAA |
| *Tl*GAM2-R1 | GTCTGTGAGTTGTAAGCATCCGCGCGGAGG |
| *Tl*GAM2-F2 | CGTCTGTGAGTTGTAAGCATCCGCGCGGA |
| *Tl*GAM2-R2 | TTAATTCGCGGCCGCCCCTTGAGGCACCGT |
| *Tl*GAM3-F1 | GCTTACGTAGAATTCGCACCACATCCCACGGAA |
| *Tl*GAM3-R1 | GTTGCTGTTGGTGTAGTATTCGGCCGCGCT |
| *Tl*GAM3-F2 | GCGCGGCCGAATACTACACCAACAGCAACC |
| *Tl*GAM3-R2 | TTAATTCGCGGCCGCCTACCTCCAACTATC |
| *Tl*GAM4-F1 | GCTTACGTAGAATTCGCACCACATCCCACGGAA |
| *Tl*GAM4-R1 | ATCACTCTCCCAGACGATGGTCCCGTCCGT |
| *Tl*GAM4-F2 | ACGGGACCATCGTCTGGGAGAGTGATCCG |
| *Tl*GAM4-R2 | TTAATTCGCGGCCGCCCCTTGAGGCACCGT |
| *Tl*GAM5-F1 | GCTTACGTAGAATTCGCACCACATCCCACGGAA |
| *Tl*GAM5-R1 | GCTGGGACCGTGTACGACCGGTTCGGGTCGTCCTCCCAGAC |
| *Tl*GAM5-R2 | ATCGTCAAGAATGGCGGTAGTCTGCCCACAGTACGCTGGGA |
| *Tl*GAM5-R3 | TTAATTCGCGGCCGCCTACCTCCAACTATC |
| S589D-F1 | AGTGTGGTCTGGGAGGATGATCCGAACA |
| S589D-R1 | ATCCTCCCAGACCACACTGCCATCCGGC |
| Q599A-F1 | TCCTATACGGTGCCTGCTGGGTGTGGCG |
| Q599A-R1 | AGCAGGCACCGTATAGGACCTGTTCGGA |
| G600Y-F1 | TATACGGTGCCTCAATACTGTGGCGTGA |
| G600Y-R1 | GTATTGAGGCACCGTATAGGACCTGTTCG |
| V603Q-F1 | CCTCAAGGGTGTGGCCAGACGACTGCGA |
| V603Q-R1 | CTGGCCACACCCTTGAGGCACCGTATAG |
| T607I-F1 | GCGTGACGACTGCGATTGTGAATGATA |
| T607I-R1 | AATCGCAGTCGTCACGCCACACCCTTG |
| V608I-F1 | TGACGACTGCGACGCTTAATGATAGTT |
| V608I-R1 | AAGCGTCGCAGTCGTCACGCCACACCCT |
| N609D-F1 | GTGACGACTGCGACGGTGGATGATAGTTG |
| N609D-R1 | ATCCACCGTCGCAGTCGTCACGCCACA |
| R613Q-F1 | GTGAATGATAGTTGGCAGTAGGCGGCC |
| R613Q-R1 | CTGCCAACTATCATTCACCGTCGCAGTC |
| QG599600AY-F1 | CCTATACGGTGCCTGCTTACTGTGGCGTGA |
| QG599600AY-R1 | GTAAGCAGGCACCGTATAGGACCTGTTCGGATC |
| TVN607-609TLD-F1 | CGTGACGACTGCGATTCTTGACGATAGTTGGAGG |
| TVN607-609TLD-R1 | GTCAAGAATCGCAGTCGTCACGCCACACCCTTGA |
| D589S-F1 | AGTGTGGTCTGGGAGAGTGACCCGAAC |
| D589S-R1 | ACTCTCCCAGACCACACTGCCATCCGGCT |
| A599Q-F1 | TCGTACACGGTCCCACAATACTGTGGGCA |
| A599Q-R1 | TTGTGGGACCGTGTACGACCGGTTCGGGT |
| G600Y-F1 | TACACGGTCCCAGCGGGGTGTGGGCAGA |
| G600Y-R1 | CCCCGCTGGGACCGTGTACGACCGGTT |
| V603Q-F1 | CCCCGCTGGGACCGTGTACGACCGGTT |
| V603Q-R1 | CACCCCACAGTACGCTGGGACCGTGTAC |
| T607I-F1 | GGCAGACTACCGCCACGCTTGACGATAGT |
| T607I-R1 | CGTGGCGGTAGTCTGCCCACAGTACGCT |
| V608I-F1 | CAGACTACCGCCATTGTGGACGATAGTTG |
| V608I-R1 | CACAATGGCGGTAGTCTGCCCACAGTAC |
| N609D-F1 | ACTACCGCCATTCTTAATGATAGTTGGCA |
| N609D-R1 | ATTAAGAATGGCGGTAGTCTGCCCACAGT |
| R613Q-F1 | TCTTGACGATAGTTGGAGGTAGGCGGCCG |
| R613Q-R1 | CCTCCAACTATCGTCAAGAATGGCGGTA |

*The restriction sites were underlined.

**Table S2** qRT-PCR primers used in this study.

| **Primers** | **Sequences (5'→3')*** |
| --- | --- |
| ARG4F | GATATGCAAGAGGATAAGGAGC |
| ARG4R | TCCTCCGGTGGCAGTTCTT |
| HAC1F | CGACTACATTACTACAGCTCCATCA |
| HAC1R | TGCTGTAATGTGTAAAGATGAATCC |
| KAR2F | TCAAAGACGCTGGTGTCAAG |
| KAR2R | TATGCGACAGCTTCATCTGG |
| PDI1F | GCCCGTTAAATTCGGTAAGCA |
| PDI1R | TCAGCTCGGTCACATCTTTG |
| ERO1F | GTTGGAAAAGCCGCATATAAACAAAACA |
| ERO1R | CAGCTTGGGCAAAGTCCTGTAAGAGTTC |
| CNE1F | CCATTGCGAATTGTCGACCC |
| CNE1R | CAACCCATTCGCCATCCTCT |
| *Tl*GAF | GCATTGTTCAAAAATACACCCCTT |
| *Tl*GAR | TAGGCGGGGACGGAGAAATTTCTGCG |

**Table S3.** Comparison of enzymatic properties of *Tl*Ga15B-GA2 and *Re*Ga15A.

| **Enzyme** | **Specific activity (U/mg)** | ***K*_m_（mg/mL）** | ***V*_max_（μmol/min/mg）** | ***k*_cat_ /*K*_m_（mL/s/mg）** |
| --- | --- | --- | --- | --- |
| ***Tl*Ga15B-GA2** | **1054.0±12.0** | **0.29±0.02** | **1093.0±22.0** | **3982.6±46.8** |
| ***Re*Ga15A** | **1030.2±23.7** | **1.04±0.09** | **1182.0±29.5** | **1243.8±23.6** |

**Fig. S1** SDS-PAGE analysis of the recombinant *Re*Ga15A. Lanes:M, molecular mass standard; 1. the crude enzyme solution; 2. the purified recombinant *Re*Ga15A; 3. the deglycosylated *Re*Ga15A treated with Endo H


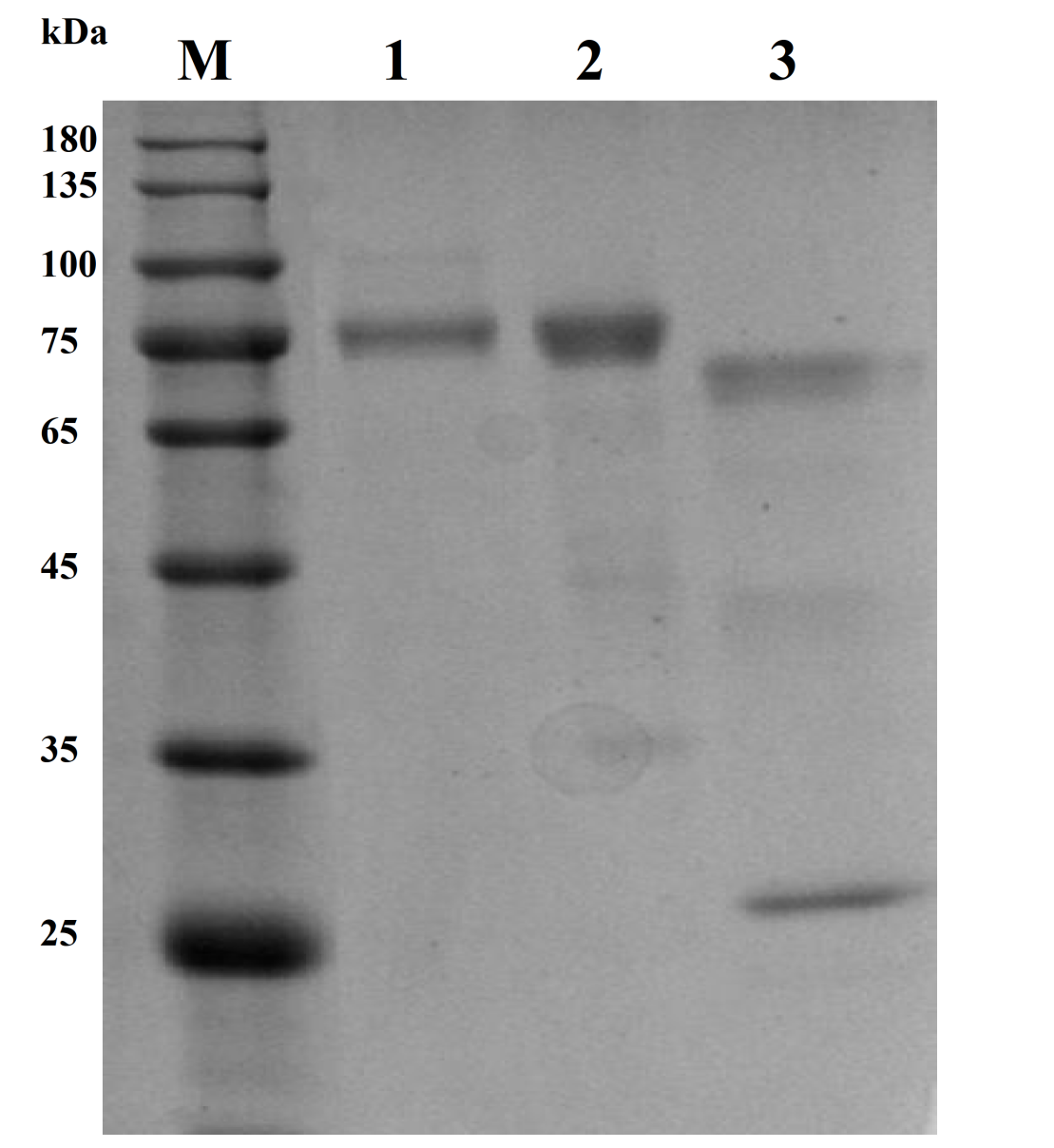


**Fig. S2** The thermostability of the purified recombinant *Re*Ga15A.


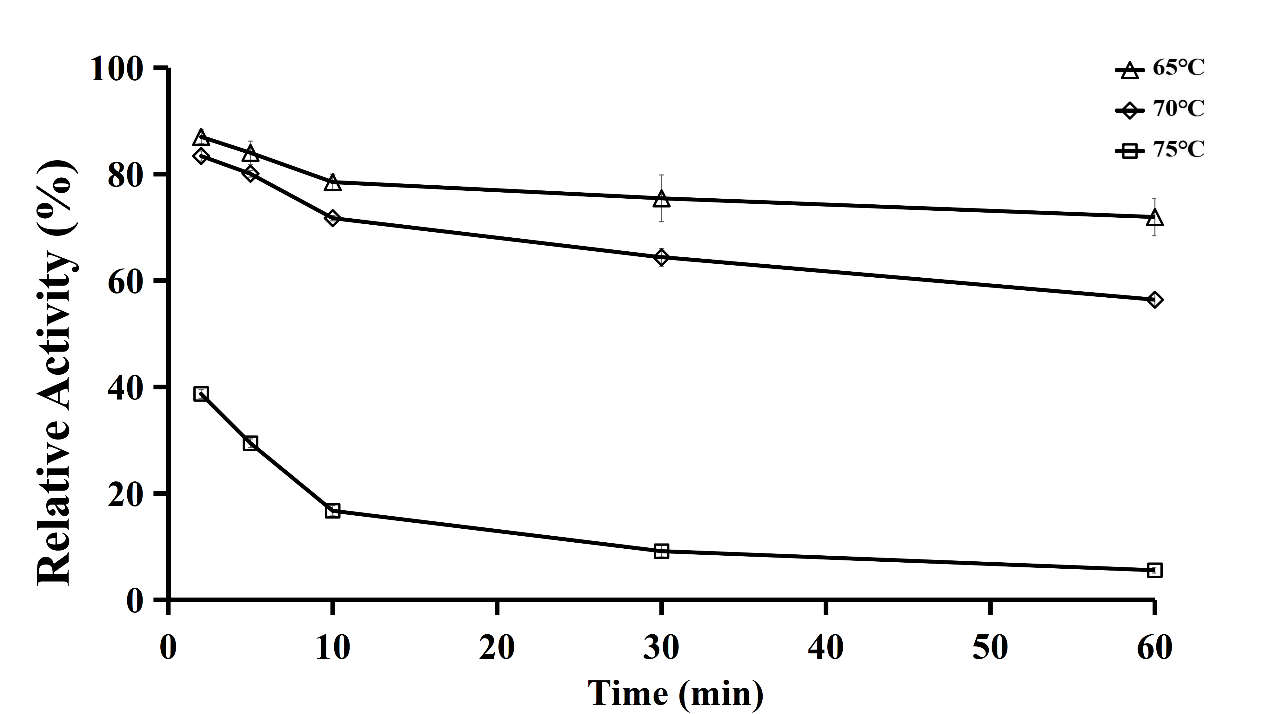


**Fig. S3** Multiple sequence alignment of *Re*Ga15A (GenBank: CAC28076.1) and *Tl*Ga15B.


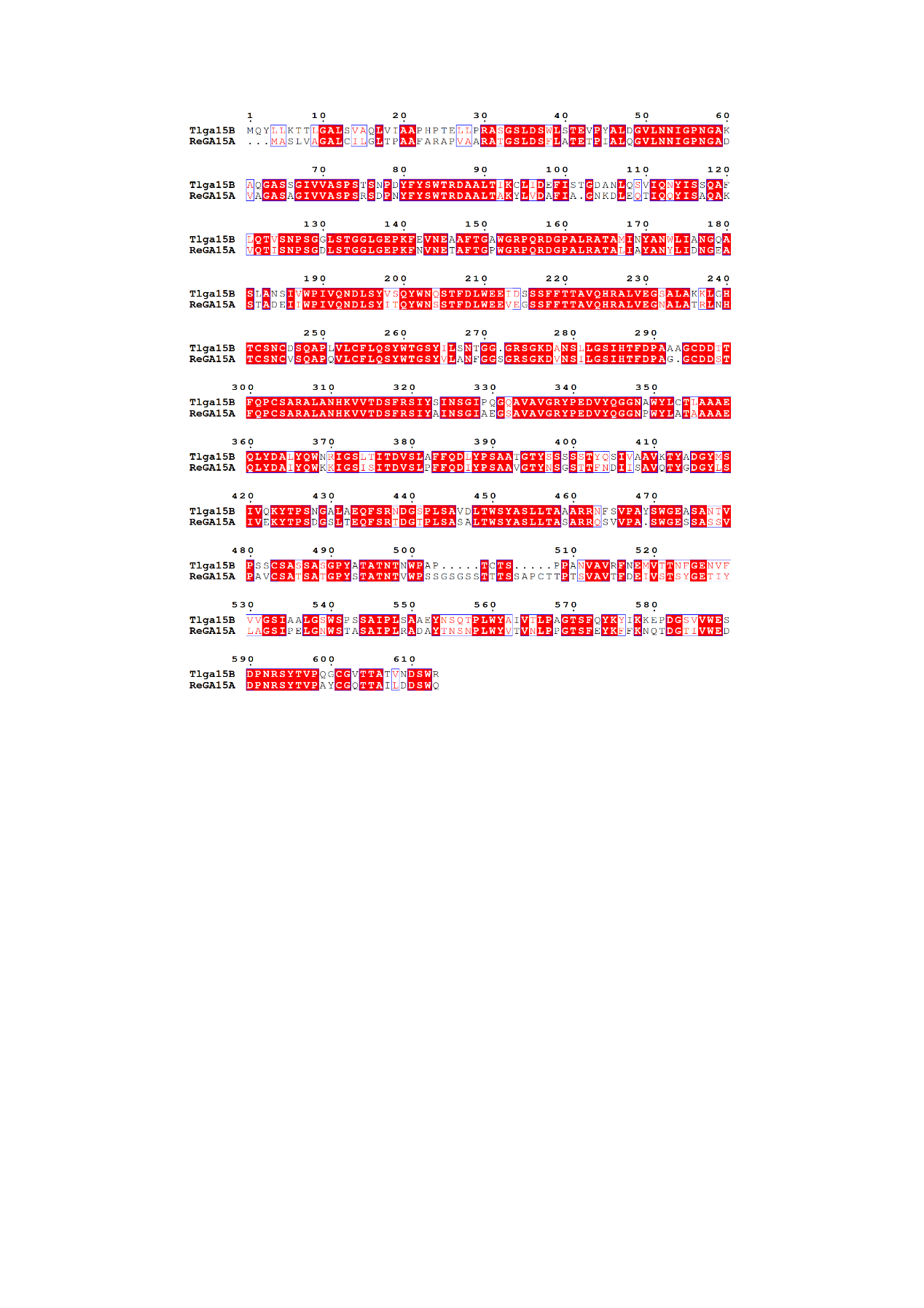


**Fig. S4** SDS-PAGE analysis of the recombinant *Re*Ga15A, *Tl*Ga15B-GA2 and mutants.

A. The expression level of single-point and adjacent position combination mutants based on *Tl*Ga15B-GA2 as template. Lane1-12, the culture supernatant of transformants WT, S589D, Q599A, G600Y, V603Q, T607I, V608L, N609D, R613Q, S589D\R613Q, Q599A\G600Y, TVN607-609ILD6. B. The expression level of single-point and adjacent position combination mutants based on *Re*Ga15A as template. Lane1-9, the culture supernatant of transformants WT and mutants.


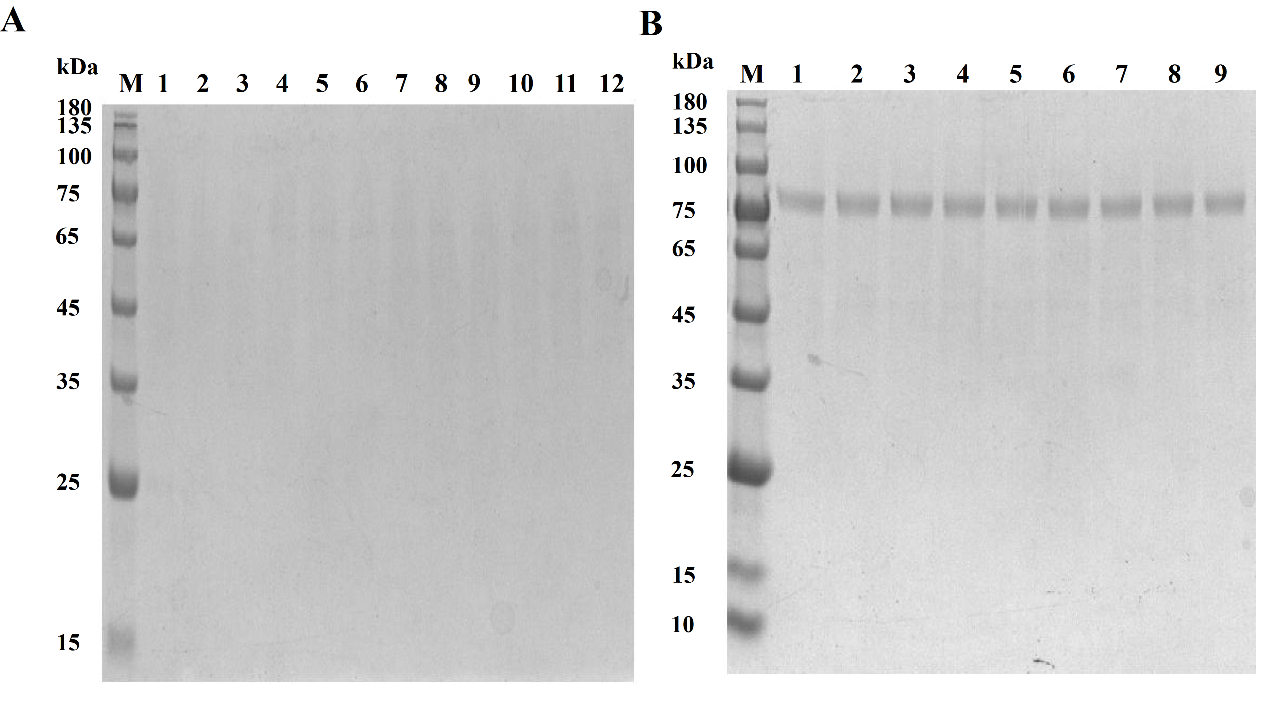


**Fig. S5** MD simulation analysis of *Tl*Ga15B-GA2 and mutants. (A) Comparison of RMSD value between WT and mutant, which is calculated from a 20ns MD simulation at 300K. (B) Comparison of normalized RMSF between WT and mutant. (c)(d) C132/C492 disulfide bond structure is shown in *Tl*Ga15B-GA2 and mutant *Tl*GA-M4.


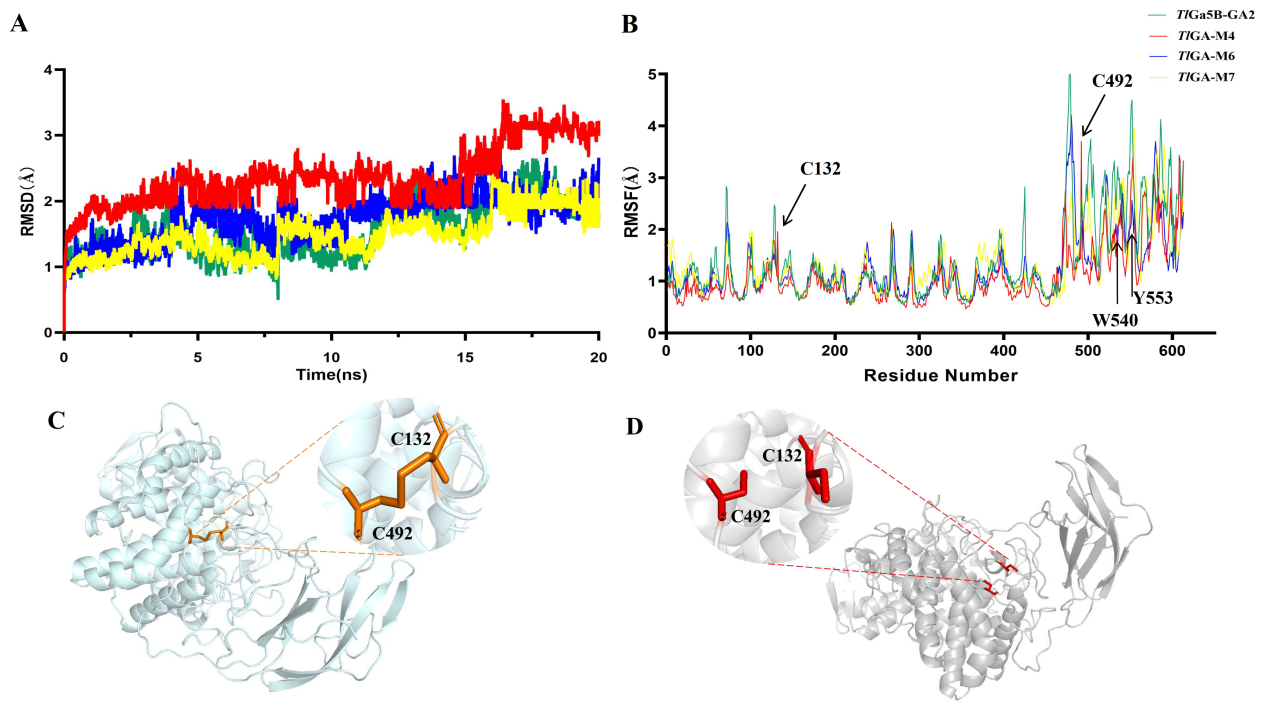

Supplement: Supplementary file 1 — Additional file 1: Table S1. Primers used in this study. Table S2. qRT-PCR primers used in this study. Table S3. Comparison of enzymatic properties of TlGa15B-GA2 and ReGa15A. Fig. S1. SDS-PAGE analysis of the recombinant ReGa15A. Fig. S2. The thermostability of the purified recombinant ReGa15A. Fig. S3. Multiple sequence alignment of ReGa15A (GenBank: CAC28076.1) and TlGa15B. Fig. S4. SDS-PAGE analysis of the recombinant ReGa15A, TlGa15B-GA2 and mutants. Fig. S5. MD simulation analysis of TlGa15B-GA2 and mutants. [file 12934_2022_1833_MOESM1_ESM.docx]
